# Improvement of Electro-Caloric Effect and Energy Storage Density in BaTiO_3_-Bi(Zn, Ti)O_3_ Ceramics Prepared with BaTiO_3_ Nano-Powder

**DOI:** 10.3390/ma17133146

**Published:** 2024-06-27

**Authors:** Geun-Soo Lee, Jeong-Seog Kim, Chae-Il Cheon

**Affiliations:** 1 Department of Materials Science & Engineering, Hoseo University, Asan 31499, Republic of Korea; aun_u@naver.com (G.-S.L.); kimjungs@hoseo.edu (J.-S.K.); 2 Department of Electronic Materials Engineering, Hoseo University, Asan 31499, Republic of Korea

**Keywords:** BaTiO_3_-Bi(Zn,Ti)O_3_, solid solution, ceramic, ferroelectric, electrocaloric, energy storage density

## Abstract

BaTiO_3_-Bi(Zn,Ti)O_3_ (BT-BZT) ceramics have been used as capacitors due to their large dielectric permittivity and excellent temperature stability and are good candidates for lead-free materials for electrocaloric and energy storage devices. However, BT-BZT ceramics often suffer from inferior properties and poor reproducibility due to heterogeneous compositional distribution after calcination and sintering. In this work, (1−*x*)BT-*x*BZT ceramics (*x* = 0~0.2) were fabricated with nano-sized BaTiO_3_ raw materials (nano-BT) by a solid-state reaction method to enhance the chemical homogeneity. The (1−*x*)BT-*x*BZT ceramics prepared from the nano-BT showed larger densities and more uniform microstructures at the lower calcination and sintering temperatures than the samples prepared from more frequently used micrometer-sized raw materials BaCO_3_, TiO_2_, Bi_2_O_3_, and ZnO. The (1−*x*)BT-*x*BZT ceramic prepared from the nano-BT displayed a phase transition from a tetragonal ferroelectric to a pseudo-cubic relaxor in a narrower composition range than the sample prepared from micro-sized raw materials. Larger adiabatic temperature changes due to the electro-caloric effect (ΔT_ECE_) and recoverable energy storage density (*U*_rec_) were observed in the samples prepared from the nano-BT due to the higher breakdown electric fields, the larger densities, and uniform microstructures. The 0.95BT-0.05BZT sample showed the largest ΔTECE of 1.59 K at 80 °C under an electric field of 16 kV/mm. The 0.82BT-0.18BZT sample displayed a *U*_rec_ of 1.45 J/cm^2^, which is much larger than the previously reported value of 0.81 J/cm^2^ in BT-BZT ceramics. The nano-BT starting material produced homogeneous BT-BZT ceramics with enhanced ECE and energy storage properties and is expected to manufacture other homogeneous solid solutions of BaTiO_3_ and Bi-based perovskite with high performance.

## 1. Introduction

Relaxor ferroelectrics have diffuse phase transitions with broad dielectric maximums that shift to lower temperatures as the measurement frequency decreases because they have local polarizations in polar nano-regions (PNRs). By comparison, normal ferroelectrics have a long-range-ordered polarization in macroscopic domains [1,2,3]. Relaxor ferroelectrics could be applied as dielectric capacitors with large capacitance and stable temperature characteristics due to their large dielectric permittivity of 10^4^~10^5^ over a broad temperature range. A very high piezoelectric coefficient d_33_ of 1500–2500 pC/N has been observed in Pb(Mg_1/3_Nb_2/3_)O_3_–PbTiO_3_-based relaxor crystals and is expected to result in groundbreaking performance improvements across various piezoelectric devices [4,5]. Recently, relaxor ferroelectrics have attracted significant attention as promising candidates for electrocaloric materials and energy storage capacitors owing to their large polarizations, slim hysteresis loops, and near-zero remnant polarizations [6,7,8].

BaTiO_3_-BiMO_3_ (M = Fe, Sc, Zn_1/2_Ti_1/2_, Mg_1/3_Nb_2/3_, ⋯) ceramics have been researched as dielectric capacitors that are operated at elevated temperatures and under a large electric field [9,10]. The crystal structure of BaTiO_3_-BiMO_3_ solid solutions changes from a ferroelectric tetragonal for BaTiO_3_ to a relaxor pseudo-cubic BaTiO_3_-BiMO_3_ solid solution when the BiMO_3_ content increases. In recent years, large electrocaloric effects and high reversible energy densities have been reported in some BaTiO_3_-BiMO_3_ ceramics [11,12,13,14,15,16,17]. Large (1.21 K) adiabatic temperature change was reported (at 143 °C) when an electric field of 5.5 kV/mm was applied to a 0.98BaTiO_3_-0.02Bi (Mg_1/2_Ti_1/2_)O_3_ ceramic [15]. This effect was attributed to an electrocaloric effect. The BaTiO_3_-Bi (Zn_1/2_Ti_1/2_)O_3_ ceramic showed a large discharge storage density of 0.81 J/cm^3^ under 12 kV/mm at the composition of 0.14 Bi(Zn_1/2_Ti_1/2_)O_3_. Large discharge energy densities of over 2 J/cm^3^ and high energy efficiencies have been reported in BaTiO_3_-Bi(Ni_1/3_Nb_2/3_)O_3_ and BaTiO_3_-Bi(Zn_1/2_Zr_1/2_)O_3_ relaxor ferroelectrics [16,17].

However, it has been previously reported that the BaTiO_3_-BiMO_3_ ceramics often showed chemical heterogeneity after the calcination and/or sintering process [18,19]. While the sintering temperature of BaTiO_3_ decreases to 1300~1100 °C by adding Bi-based perovskite compounds, the solid solution reaction of the BaTiO_3_-BiMO_3_ ceramic during a calcination process was reported to be completed at temperatures as high as 950 °C via several intermediate compounds such as BaBi_4_Ti_4_O_15_, BaBiO_3,_ and Bi_4_Ti_3_O_12_ [19,20,21]. The chemical heterogeneity frequently led to multicolored powders after calcination and/or heterogeneous microstructures after sintering in the BaTiO_3_-Bi(M)O_3_ ceramics and is expected to result in inferior electrical properties like low breakdown electric field [19,22].

In this work, the electrocaloric effect and the energy storage density of a BaTiO_3_-Bi(Zn_1/2_Ti_1/2_)O_3_ (BT-BZT) solid solution ceramic was investigated. In order to reduce the chemical heterogeneity during manufacturing, the BT-BZT ceramics were prepared by a conventional ceramic process with a nano-sized BaTiO_3_ powder instead of the micron-sized BaCO_3_ and TiO_2_ powders which are the commonly used raw materials for BaTiO_3_. Samples with the same compositions were also prepared with micrometer-sized BaCO_3_ and TiO_2_ powders for comparison. The changes in crystal structure, microstructure, and dielectric phase transition behavior with increasing BZT mole fraction were investigated in BT-BZT ceramics. The electrocaloric effect (ECE) and energy storage characteristics were compared in the BT-BZT ceramics prepared from the nano-sized BaTiO_3_ and the micron-sized raw materials.

## 2. Experimental

(1−*x*)BaTiO_3_-*x*Bi(Zn_1/2_Ti_1/2_)O_3_ ((1−*x*)BT-xBZT) ceramics (*x* = 0~0.2) were fabricated with raw materials of nano-sized BaTiO_3_ (<100 nm, ≥99%, Sigma Aldrich, St. Louis, MO, USA), Bi_2_O_3_ (≥99.9%, Sigma Aldrich), ZnO (≥99.9%, High Purity Chemicals Co., Navi Mumbai, India), and TiO_2_ (≥99.9%, High Purity Chemicals Co.) by a solid-state reaction method. We hereafter refer to these samples as “nano-samples”. For comparison, we prepared the other samples, which were denominated as “micro-samples”, with the more frequently used raw materials, micrometer-sized BaCO_3_ (≥99%, Sigma Aldrich), TiO_2_ (≥99.9%, High Purity Chemicals Co.), Bi_2_O_3_, and ZnO (≥99.9%, High Purity Chemicals Co.). The raw materials were mixed with yttria-stabilized zirconia balls in ethanol by ball milling. After drying the mixed slurry, the micro-samples were calcined in two steps: first at 850 °C for 5 h and second at 950 °C for 3 h. The nano-samples were calcined in a single step at 850 °C for 2 h. The calcined powders were pulverized by a ball mill and 1 wt.% polyvinyl butyral (PVB) binder was added during the milling. The granulated powders were formed into a disk shape by uniaxial pressing. The compacted nano-samples were sintered at 1200 °C for 2 h and the micro-samples at 1225–1320 °C for 2 h after binder burn-out at 600 °C for 2h. Microstructures and crystal structures were analyzed using a scanning electron microscope (SEM) (SNE-4500M, SEC, Cheongju-si, Republic of Korea) and X-ray diffraction (XRD) (XD-D1, Shimatzu, Kyoto, Japan). Silver electrodes were painted on the surfaces of the sintered samples and fired at 800 °C for 15 min for electrical measurements.

The changes in dielectric properties with temperature were measured at frequencies of 1 kHz–1 MHz using an impedance analyzer (4294A, Agilent, Santa Clara, CA, USA). The ferroelectric polarization–electric field (P-E) hysteresis loops were measured at temperatures ranging from −60 °C to 150 °C in silicon oil using a ferroelectric tester (RT66A, Radiant Co., Ltd., Bucheon-si, Republic of Korea) and a high voltage amplifier (Trek, 609E-6-L-CE, Miami, FL, USA). An adiabatic temperature change, due to ECE (Δ*T_ECE_*), was calculated indirectly using the thermodynamic Equation (1) and Maxwell’s relation (2) [23].

(1) $$∆T_{ECE}=-\int_{E_{1}}^{E_{2}}\frac{T}{\rho C}\cdot {\left(\frac{\partial P}{\partial T}\right)}_{E}dE$$

(2) $${\left(\frac{\partial S}{\partial E}\right)}_{T}={\left(\frac{\partial P}{\partial T}\right)}_{E}$$

where *T*, *ρ*, *C*, *P*, *E*, and *S* are temperature, density, heat capacity, polarization, the electric field, and entropy, respectively. In Equation (1), Δ*T_ECE_* is the temperature change due to ECE when the electric field changes from *E*_1_ to *E*_2_. The previously reported heat capacity was used in the calculation [24]. The polarizations were determined from the upper branches (*E* > 0) of the P-E hysteresis loops. Energy storage density (charge energy density, *U_st_*), recoverable energy storage density (discharge energy density, *U_rec_*), and discharge-to-charge energy efficiency (*η*) were calculated in the following Equations (3)–(5) from the P-E hysteresis curves.

(3) $$U_{st}=\int_{0}^{P_{max}}E\;dP$$

(4) $$U_{rec}=\int_{P_{r}}^{P_{max}}E\;dP$$

(5) $$\eta (\%)=\frac{U_{rec}}{U_{st}}\times 100$$

The remnant polarization (*P_r_*) and the maximum polarization (*P_max_*) were obtained from the P-E hysteresis curves.

## 3. Results and Discussion

**Figure 1:**
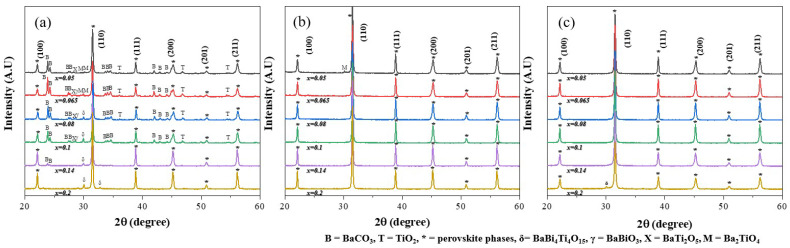
XRD patterns for the (1−*x*)BT-*x*BZT micro-samples (**a**) after the first calcination at 850 °C for 5 h, (**b**) after the second calcination at 950 °C for 2 h, and (**c**) for the nano-samples calcined at 850 °C for 2 h.

Figure 1 shows the XRD patterns for the micro-samples (a) after the first calcination at 850 °C for 5 h, (b) after the second calcination at 950 °C for 2 h, and (c) for the nano-samples calcined at 850 °C for 2 h.

The XRD patterns in Figure 1 were indexed based on a cubic lattice. The micro-samples display many XRD peaks for impurity phases after the first calcination at 850 °C for 5 h in all composition ranges of (1−*x*)BT-*x*BZT (*x* = 0.05~0.20). The impurity phases were identified as unreacted BaCO_3_ and TiO_2_, and minute amounts of intermediate barium titanate compounds (Ba_2_TiO_4_ and BaTi_2_O_5_) in the 0.95BT-0.05BZT sample. Unreacted BaCO_3_ was a major impurity phase in the (1−*x*)BT-*x*BZT sample with *x* = 0.05~0.1 and disappeared when the mole fraction of BZT (*x*) was increased to 0.2. This result agrees with previous reports that unreacted BaCO_3_ was observed at high temperatures of 900 °C in the calcination process of BaTiO_3_ and disappeared at lower calcination temperatures when the amount of BiMO_3_ increased in BaTiO_3_-BiMO_3_ solid solutions. When the mole fraction of the BZT phase increased, the major intermediate phase changed to BaBi_4_Ti_4_O_15_ and the other impurity phases were not observed in the 0.8BT-0.2BZT sample after the first calcination. As shown in Figure 1b, most unreacted and intermediate phases disappeared after the second calcination at 950 °C for 2 h in the micro-samples. On the other hand, the nano-samples show XRD patterns for a single perovskite after a single-step calcination at 850 °C for 2 h. A minute amount of an intermediate BaBi_4_Ti_4_O_15_ phase was observed in the 0.8BT-0.2BZT nano-sample. Figure 1 demonstrates that the BT nano-powder as a starting material facilitates the homogeneous reaction of the BT-BZT solid solution and reduces the calcination temperature by 100 °C. To examine the effect of the synthesis route on the BT-BZT reaction during calcination, we analyzed the phase evolution when the micrometer-sized BaTiO_3_ powder (<2 µm, Sigma Aldrich) was used as a starting material. Many diffraction peaks for impurity phases were observed after the first calcination at 850 °C for 5 h and nearly disappeared after the second calcination at 950 °C for 2 h, similar to the micro-samples (Figure S1). The calcined powders were imaged by SEM (Figure S2). Figure S2 shows that the particle size of the nano-sample was much smaller than the micro-sample, although it is difficult to accurately determine the particle size from Figure S2 due to agglomeration. Therefore, the smaller particle size of the nano-sample is considered to lead to a more homogeneous reaction at a lower calcination temperature than the micro-sample.

**Figure 2:**
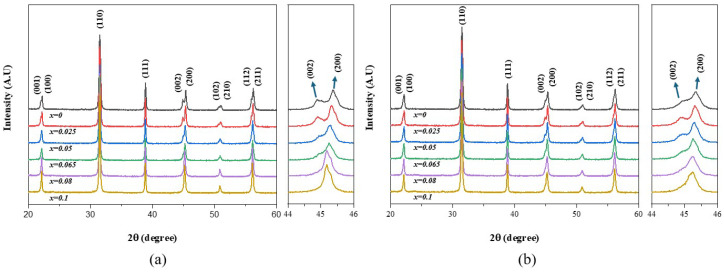
The XRD patterns of the sintered (1−*x*)BT-*x*BZT ceramics: (**a**) nano-samples and (**b**) micro-samples.

The XRD patterns of (a) the nano-samples and (b) the micro-samples after sintering are shown in Figure 2. The detailed XRD patterns for the (200) diffraction peak at 2θ = 44~46° were also included to clarify the crystal structure change.

As shown in Figure 2, the tetragonal (002)_T_ and (200)_T_ diffraction peaks merged to a single pseudo-cubic (200)_pC_ diffraction peak around *x* = 0.08 when the BZT mole fraction (*x*) increased from 0 to 0.1 in both nano- and micro-samples. All samples with a BZT mole fraction larger than 0.1 (0.1 < *x* ≤ 0.2) had a single (200)_pC_ diffraction peak and very small diffraction peaks for impurity phases were observed in micro-samples (Figure S3). This result is consistent with the previous report that the crystal structure changes from a ferroelectric tetragonal for BaTiO_3_ (*x* = 0) to a relaxor-like pseudo-cubic around *x* = 0.08 when the BZT mole fraction increased in the (1−*x*)BT-*x*BZT ceramics [13]. Figure 2 displays that the (200)_pC_ diffraction peaks for the micro-samples with *x* = 0.08~0.1 are more asymmetrical than the (200)_pC_ diffraction peak for the nano-samples. The crystal structures of the (1−*x*)BT-*x*BZT ceramics (*x* = 0.065~0.1) were analyzed in detail by the Rietveld refinement method and the results are shown in Table 1. The X-ray diffraction patterns were refined based on the two-phase model consisting of a tetragonal (P4mm) phase and a cubic (Pm-3m) phase. The Rietveld refinement profiles are shown in Figure S4. When the BZT fraction increased from 0.065 to 0.08, the fractions of the tetragonal phase decreased more slowly from 0.695 to 0.306 in the micro-samples, while the fraction of the tetragonal phase decreased from 0.716 to 0.135 in the nano-samples. This result suggests that the phase transition from a tetragonal to a pseudo-cubic occurred over a wide composition range in the micro-sample. The nano-samples had larger densities and more uniform microstructures than the micro-samples (Figures S5 and S6). The average grain size increased slightly as the BZT mole fraction increased to 0.2 in the nano-sample (Figure S6). The micro-samples showed similar microstructures but had some non-uniform grains (Figure S6).

**Figure 3:**
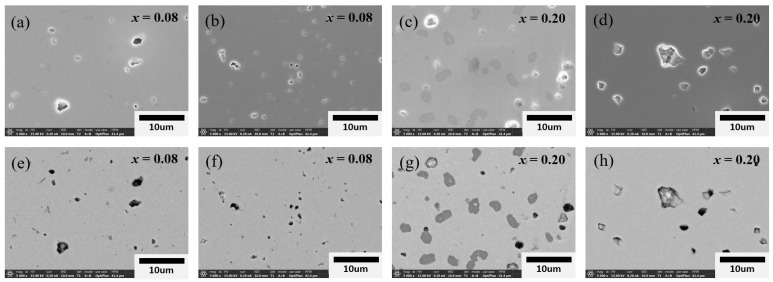
Secondary electron (SE) images (**a**–**d**) and backscattered electron (BSE) images (**e**–**h**) of the polished surface in (1−*x*)BT-*x*BZT ceramics: (**a**,**e**) *x* = 0.08 micro-sample, (**b**,**f**) *x* = 0.08 nano-sample, (**c**,**g**) *x* = 0.2 micro-sample, and (**d**,**h**) *x* = 0.2 nano-sample.

Chemical heterogeneity in 0.8BT-0.2BZT ceramics has previously been observed through backscattered electron (BSE) images and an energy dispersive spectroscopy (EDS) analysis [19]. Figure 3 shows the secondary electron (SE) images (a~d) and BSE images (e~h) of the polished surfaces of 0.92BT-0.08BZT and 0.8BT-0.2BZT ceramics. Heterogeneous areas with dark contrasts are more clearly observed in the BSE images. These BSE images show that the micro-samples contain heterogeneous areas that are wider and larger in number than that of the nano-samples. Because the areas with heavy elements have bright contrast in the BSE image, the dark heterogeneous areas are believed to be deficient in heavy elements such as Bi, as previously reported. The EDS analysis shows that the dark areas had a different chemical composition from the bright matrix area; in particular, the Bi content was very low (Figures S7 and S8). The difference in the Bi content was much larger in the micro-samples, as shown in Figure S7 and S8. This result indicates that the composition heterogeneity of the micro specimen is more severe and that the compositional heterogeneity of the BT-BZT solid solution is greatly reduced when BT nano-powder is used as a starting material.

**Figure 4:**
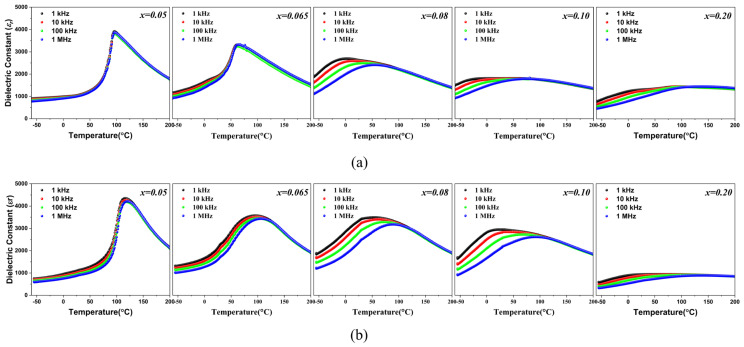
Temperature-dependent dielectric permittivities of (1−*x*)BT-*x*BZT ceramics: (**a**) nano-samples and (**b**) micro-samples.

Figure 4 shows the temperature-dependent dielectric permittivities of the (1−*x*)BT-*x*BZT ceramics. The frequency independent dielectric peaks are observed in the nano-samples with a BZT mole fraction (*x*) of 0.05 and 0.065 and the micro-samples show slight frequency dispersions of the dielectric peaks at *x* = 0.05 and 0.065. The samples with *x* = 0.08~0.2 show broad dielectric peaks with distinct frequency dispersion that the temperature for the maximum dielectric constant (*T*_max_) shifted to low temperature when the measuring frequency decreased. This broad dielectric maximum with frequency dispersion is known to result from the phase transition of a relaxor ferroelectric [25,26]. Table 1 demonstrate that a phase transition occurred in the (1−*x*)BT-*x*BZT ceramics from a ferroelectric with a tetragonal structure to a relaxor with a pseudo-cubic structure around *x* = 0.08 at room temperature, which is consistent with previous reports [13]. Figure 4 shows that *T*_max_ shifts towards room temperature and the dielectric peak becomes broader when the BZT mole fraction increases. The micro-samples show slight frequency dispersions even at *x* = 0.05 and 0.065 and broader dielectric peaks than the nano-samples with the same compositions. These results indicate that the phase transition is more diffuse in the micro-sample due to the inhomogeneous composition distribution. The gradual phase transition depicted in Figure 2 and Table 1 gives rise to the dielectric behavior of the micro-samples presented in Figure 4.

**Figure 5:**
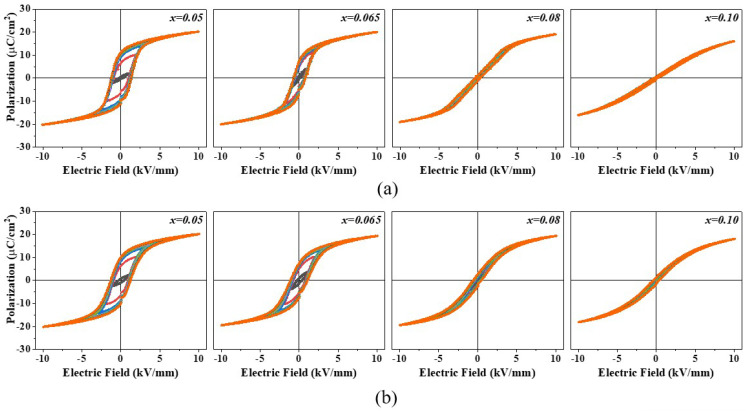
The P-E hysteresis curves of (1−*x*)BT-*x*BZT ceramics measured at room temperature: (**a**) nano-samples and (**b**) micro-samples.

Figure 5 shows the P-E hysteresis curves of (1−*x*)BT-*x*BZT ceramics measured at room temperature. The P-E curves with a maximum applied electric field ranging from 1 kV/mm to 10 kV/mm are plotted on the same graph in Figure 5. The samples with *x* = 0.05 and 0.065 show well-saturated ferroelectric P-E hysteresis curves and the P-E curve became slanted and slim, which is typical for a relaxor ferroelectric at *x* = 0.08 and *x* = 0.1. This result supports that the (1−*x*)BT-*x*BZT ceramics have a ferroelectric phase at *x* < 0.08 and the phase change to a relaxor phase occurred at around *x* = 0.08. The P-E hysteresis curves of (1−*x*)BT-*x*BZT ceramics with *x* = 0.1~0.2 were similar shapes to that of the 0.9BT-0.1BZT sample. The P-E hysteresis curve of the micro-sample was more slanted at *x* = 0.05~0.065 than that of the nano-samples and resembles the mixed P-E curve of a ferroelectric and a relaxor at *x* = 0.08. This result also indicates that the phase transition from a ferroelectric to a relaxor occurred more gradually in the micro-samples.

**Figure 6:**
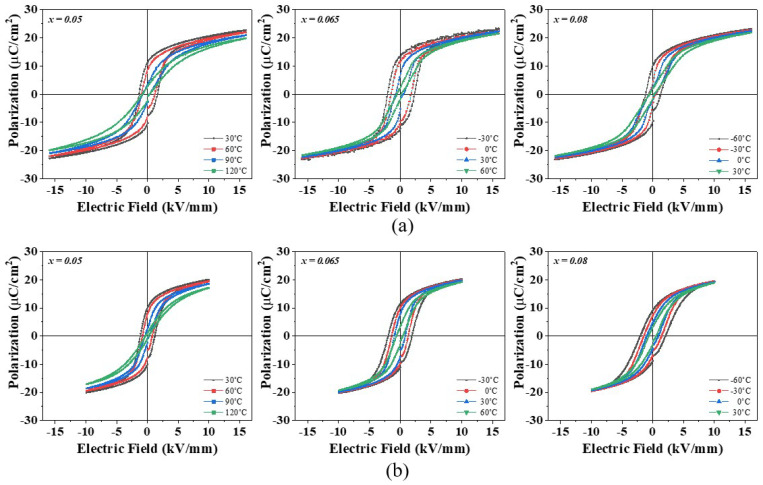
The changes of the P-E hysteresis curves with temperature in the (1−*x*)BT-*x*BZT ceramics: (**a**) nano-samples and (**b**) micro-samples.

The changes in the P-E hysteresis curves with temperature were measured in the temperature range of −60 °C to 150 °C. The changes in the P-E hysteresis curves close to *T*_max_ in Figure 4 were shown in Figure 6. A larger electric field could be applied to the nano-samples (16 kV/mm) than the micro-samples (10 kV/mm) because of their larger densities and more uniform microstructures. The nano-samples with *x* = 0.05 and 0.065 had well-saturated ferroelectric P-E hysteresis curves at temperatures lower than *T*_max_. As the temperature increased, a remanent polarization (*P*_r_) and a coercive electric field (*E*_C_) decreased continuously, and the P-E curve became slim around 90 °C at *x* = 0.05 and around 60 °C at *x* = 0.065 because of the phase transition from a ferroelectric to a paraelectric that occurred at that temperature. The 0.92BT-0.08BZT nano-sample shows a normal ferroelectric P-E hysteresis curve at temperatures as low as −60 °C, a double hysteresis curve with significantly decreased *P*_r_ and *E*_C_ at a temperature range of −30~0 °C, and a slim P-E curve at 30 °C. This suggests that the 0.92BT-0.08BZT nano-sample was a non-ergodic relaxor state at −60 °C, which was transformed to a ferroelectric state by an applied electric field. In addition, this suggests that a phase change to an ergodic relaxor state occurred above −60 °C. The transition temperatures observed from the P-E hysteresis curves in Figure 6 match well with *T*_max_ in Figure 4. The P-E curves showed a similar trend of change with respect to temperature for both microsamples and nano-samples, however, this change was more gradual in the first case.

**Figure 7:**
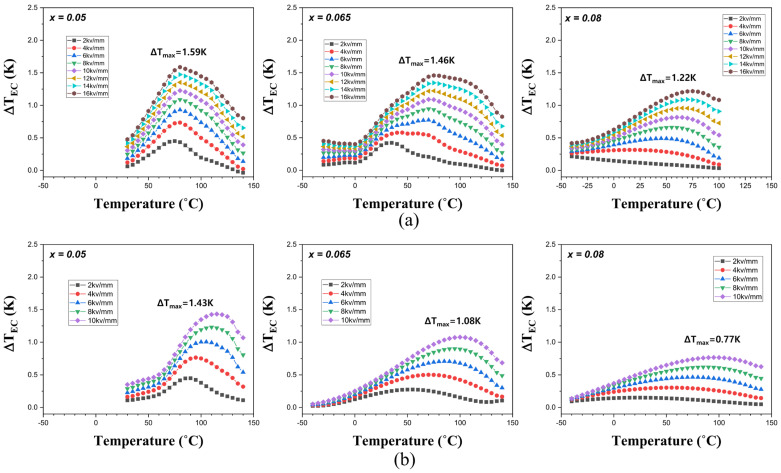
Changes in Δ*T*_ECE_ in the (1−*x*)BT-*x*BZT ceramics with increasing temperature: (**a**) nano-samples and (**b**) micro-samples.

From these temperature changes in the P-E hysteresis curves, the adiabatic temperature changes due to ECE (Δ*T*_ECE_) were calculated indirectly from the P-E hysteresis curves at the maximum electric field of 16 kV/mm in the nano-samples and 10 kV/mm in the micro-samples. The Δ*T*_ECE_ of the (1−*x*)BT-*x*BZT ceramics with *x* > 0.08 are not shown in Figure 7 because of very weak electrocaloric effects. The maximum Δ*T*_ECE_ (Δ*T*_max_) in Figure 7 is observed at a temperature close to *T*_max_ in Figure 4. When the BZT mole fraction was increased, the temperature for Δ*T*_max_ (*T*_maxEC_) decreased and the Δ*T*_ECE_ peaks became broader. The nano-samples showed a larger Δ*T*_maxEC_ than the micro-samples with the same composition, mainly because a larger electric field was applied. The *T*_maxEC_, the applied electric field (Δ*E*), and the Δ*T*_max_ in this work are compared with previously reported BaTiO_3_-BiMO_3_ ceramics in Table 2. The 0.95BT-0.05BZT nano-sample in this work shows the largest Δ*T*_max_ of 1.59 K at 80 °C under an electric field of 16 kV/mm.

**Figure 8:**
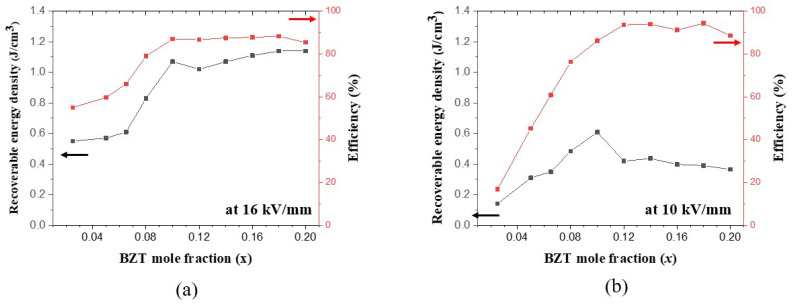
Recoverable energy density and discharge-to-charge energy efficiency as a function of the BZT mole fraction in the (1−*x*)BT-*x*BZT ceramics: (**a**) nano-samples and (**b**) micro-samples.

Recoverable energy storage density (*U*_rec_) and discharge-to-charge energy efficiency (*η*) were calculated from the P-E hysteresis curves at the maximum electric field of 16 kV/mm in the nano-samples and 10 kV/mm in the micro-samples. Figure 8 shows that both *U*_rec_ and *η* of the (1−*x*)BT-*x*BZT ceramics were very low in the ferroelectric phase with *x* < 0.08 and greatly enhanced in the relaxor phase with *x* ≥ 0.08. It has been reported that a relaxor ferroelectric phase with a small *P*_r_ and a low hysteresis loss has a large recoverable energy density and a high energy efficiency, while a ferroelectric phase with a large *P*_r_ and a large hysteresis loss has a small recoverable energy density and a low efficiency. The (1−*x*)BT-*x*BZT ceramics with *x* > 0.1 had larger breakdown electric fields than the samples with *x* ≤ 0.1 likely due to their larger densities and uniform microstructures. The P-E hysteresis curves, the maximum *U*_rec_ and *η* in the nano-samples at the applied electric field of 20 kV/mm and in the micro-samples at 13 kV/mm are shown in Figures S9 and S10.

The maximum *U*_rec_, *η,* and breakdown strengths (BDS) of the (1−*x*)BT-*x*BZT are compared with the reported values in other BaTiO_3_-BiMO_3_ ceramics in Table 3. The *U*_rec_ and *η* of the (1−*x*)BT-*x*BZT nano-sample were 1.33 J/cm^2^ and 87.6%, 1.42 J/cm^2^ and 88.2%, and 1.45 J/cm^2^ and 86.7% at *x* = 0.14, 0.16, and 0.18, respectively. The *U*_rec_ is about two times larger than the previously reported value in the 0.86BT-0.14BZT ceramic as shown in Table 3. The *η* of the nano-samples, however, is smaller than the reported values in other BaTiO_3_-BiMO_3_ ceramics, which may be because the larger electric field applied to the nano-sample led to larger ferroelectric hysteresis loss. The energy efficiencies of the micro-samples in this work were as high as 92~94.5% as shown in Figure S10. Another reason for the lower efficiency compared to previously reported BaTiO_3_-BiMO_3_ ceramics is that the energy efficiency was calculated from the bipolar P-E hysteresis curves in this work, whereas the efficiencies were calculated from the uni-polar P-E curves in previous reports. Table 3 shows that *U*_rec_ and BDS were significantly improved in the (1−*x*)BT-*x*BZT ceramics by using the BT nano-powder as a starting material. The nano-BT starting material is expected to enhance the energy storage properties in other BaTiO_3_-BiMO_3_ ceramics.

## 4. Conclusions

(1−*x*)BT-*x*BZT ceramics (*x* = 0~0.2) were fabricated with raw materials of nano-sized BaTiO_3_ using a solid-state reaction method to enhance chemical homogeneity. A BT-BZT solid solution phase was formed in nano-samples by a single step calcination at 850 °C which is 100 °C lower than the calcination temperature of the micro-samples. The tetragonal (002)_T_ and (200)_T_ diffraction peaks merged to a single pseudo-cubic (200)_pC_ diffraction peak around *x* = 0.08 when the BZT mole fraction was increased in the sintered samples. The (1−*x*)BT-*x*BZT ceramics showed frequency independent dielectric peaks at *x* ≤ 0.065 and broad dielectric peaks with the frequency dispersion at *x* = 0.08~0.2. The well-saturated ferroelectric P-E hysteresis curves were observed at room temperature in the (1−*x*)BT-*x*BZT ceramics with *x* ≤ 0.065 and the P-E curve became more slanted and slimmer at *x* ≥ 0.08. When the temperature increased from −60 °C to 150 °C, the P-E hysteresis curve changed from a well-saturated shape to a slim shape at around 90 °C for *x* = 0.05, around 60 °C for *x* = 0.065, and about 0 °C for *x* = 0.08. The micro-samples showed broader dielectric peaks, more slanted P-E hysteresis curves, and more gradual changes in the shape of the P-E curve with temperature than the nano-samples due to compositional heterogeneity.

The nano-samples showed larger adiabatic temperature changes due to ECE and recoverable energy storage density due to larger breakdown electric fields, larger densities, and more uniform microstructures than the micro-samples. The 0.95BT-0.05BZT nano-sample showed the largest ΔT_ECE_ of 1.59 K at 80 °C under an electric field of 16 kV/mm. The *U*_rec_ and *η* of the 0.82BT-0.18BZT nano-sample were 1.45 J/cm^2^ and 86.7%. The *U*_rec_ was about two times larger than those of the micro-samples and the previously reported values in BT-BZT ceramics. These results suggest that the manufacture of homogeneous BT-BZT ceramics using BT nano-powder is an effective way to enhance the electrocaloric effect and energy storage properties.

## Figures and Tables

**Table 1:** The structure refinement results for the (1−*x*)BaTiO_3_-*x*Bi(Zn_1/2_Ti_1/2_)O_3_ ceramics.

Samples	Phase	Fraction	Lattice Parameters			R-Factors				
	(SG)	(%)	a(Å)	b(Å)	c(Å)	R_p_	R_wp_	R_exp_	R_b_	R_f_
0.065BZT- nano-sample	P4mm	71.6	3.99765	3.99765	4.0282	4.58	5.87	4.18	3.28	3.08
	Pm-3m	28.4	4.00731	4.00731	4.00731				2.66	2.16
0.065BZT-micro-sample	P4mm	69.5	4.00002	4.00002	4.03058	4.49	5.69	4.32	2.21	2.67
	Pm-3m	30.5	4.00937	4.00937	4.00937				1.62	1.5
0.08BZT- nano-sample	P4mm	13.5	3.99341	3.99341	4.03986	4.38	5.54	4.12	5.19	6.63
	Pm-3m	86.5	4.00832	4.00832	4.00832				1.92	1.71
0.08BZT- micro-sample	P4mm	30.6	3.99955	3.99955	4.03276	4.49	5.66	4.26	3.11	3.55
	Pm-3m	69.4	4.01035	4.01035	4.01035				2.02	1.76
0.10BZT- micro-sample	P4mm	12.8	3.99351	3.99351	4.03937	5.62	7.12	5.79	6.89	9.15
	Pm-3m	87.2	4.00842	4.00842	4.00842				2.13	2.24
0.10BZT- nano-sample	P4mm	14.6	3.99874	3.99874	4.03586	4.43	5.62	4.24	3.75	3.91
	Pm-3m	85.4	4.01114	4.01114	4.01114				1.86	1.44

**Table 2:** Electrocaloric effects of BaTiO_3_-BiMO_3_ ceramics obtained by an indirect method. Δ*T*_max_ is the maximum adiabatic temperature change due to ECE, Δ*E* is the applied electric field, and *T*_maxEC_ is the temperature for Δ*T*_max_.

Composition	*T*_maxEC_ (°C)	Δ*E* (kV/mm)	Δ*T*_max_ (°C)	Ref
0.95BaTiO_3_-0.05Bi(Zn_0.5_Ti_0.5_)O_3_ (nano)	80	16	1.59	This work
0.95BaTiO_3_-0.05Bi(Zn_0.5_Ti_0.5_)O_3_ (micro)	115	10	1.43	This work
0.935BaTiO_3_-0.065Bi(Zn_0.5_Ti_0.5_)O_3_ (nano)	75	16	1.45	This work
0.935BaTiO_3_-0.065Bi(Zn_0.5_Ti_0.5_)O_3_ (micro)	100	10	1.08	This work
0.92BaTiO_3_-0.08Bi(Zn_0.5_Ti_0.5_)O_3_ (nano)	75	16	1.22	This work
0.92BaTiO_3_-0.08Bi(Zn_0.5_Ti_0.5_)O_3_ (micro)	95	10	0.77	This work
0.99BaTiO_3_-0.01Bi(Mg_0.5_Ti_0.5_)O_3_	138	5.5	1.02	[15]
0.98BaTiO_3_-0.02Bi(Mg_0.5_Ti_0.5_)O_3_	143	5.5	1.21	[15]
0.97BaTiO_3_-0.03Bi(Li_1/3_Ti_2/3_)O_3_	125	2.2	0.66	[14]
0.94BaTiO_3_-0.06Bi(Li_1/3_Ti_2/3_)O_3_	50	2.2	0.38	[14]

**Table 3:** Energy storage properties of BaTiO_3_-BiMO_3_ ceramics. *U*_rec_ is the recoverable energy density, *η* is the discharge-to-charge energy density, and BDS is the breakdown strength.

Composition	*U*_rec_ (J/cm^3^)	*η* (%)	BDS (kV/mm)	Ref
0.86BaTiO_3_-0.14Bi(Zn_1/2_Ti_1/2_)O_3_ (nano)	1.33	87.6	20	This work
0.84BaTiO_3_-0.16Bi(Zn_1/2_Ti_1/2_)O_3_ (nano)	1.42	88.2	20	This work
0.82BaTiO_3_-0.18Bi(Zn_1/2_Ti_1/2_)O_3_ (nano)	1.45	86.7	20	This work
0.86BaTiO_3_-0.14Bi(Zn_1/2_Ti_1/2_)O_3_ (micro)	0.66	92.5	13	This work
0.84BaTiO_3_-0.16Bi(Zn_1/2_Ti_1/2_)O_3_ (micro)	0.65	94.3	13	This work
0.82BaTiO_3_-0.18Bi(Zn_0.5_Ti_0.5_)O_3_ (micro)	0.60	93	13	This work
0.86BaTiO_3_-0.14Bi(Zn_0.5_Ti_0.5_)O_3_	0.81	94	12	[13]
0.90BaTiO_3_-0.10Bi (Mg_2/3_Nb_1/3_)O_3_	1.13	-	14.3	[11]
0.90BaTiO_3_-0.10Bi (Mg_2/3_Nb_1/3_)O_3_ + 0.3 wt% MnCO_3_	1.7	88.6	21	[12]
0.88BaTiO_3_-0.12Bi (Ni_2/3_Nb_1/3_)O_3_	2.0	95.9	20	[16]
0.85BaTiO_3_-0.15Bi(Zn_1/2_Zr_1/2_)O_3_	2.47	94.4	25	[17]

## Data Availability

The original contributions presented in the study are included in the article/Supplementary Material, further inquiries can be directed to the corresponding author.

## Supplementary Materials

The following supporting information can be downloaded at: https://www.mdpi.com/article/10.3390/ma17133146/s1.
