# Supplementary material for: Improvement of Electro-Caloric Effect and Energy Storage Density in BaTiO3-Bi(Zn, Ti)O3 Ceramics Prepared with BaTiO3 Nano-Powder
Source: Materials (Basel). 2024 Jun 27;17(13):3146. doi: 10.3390/ma17133146 (PMC11242748; doi:10.3390/ma17133146)
Supplement: Supplementary file 1 [file materials-17-03146-s001.zip › materials-3048670-supplementary.pdf]

Supplementary Materials

Improvement of Electro-caloric Effect and Energy Storage Density in $BaTiO_3$-$Bi(Zn,Ti)O_3$ Ceramics prepared with $BaTiO_3$ nano-powder

Geun-Soo Lee[1], Jeong-Seog Kim[1], and Chae Il Cheon[1,2*]
*Corresponding author : cicheon@hoseo.edu
[1]*Department of Materials Science & Engineering, Hoseo University, Asan 31499, Korea*
[2]*Department of Electronic Materials Engineering, Hoseo University, Asan 31499, Korea*

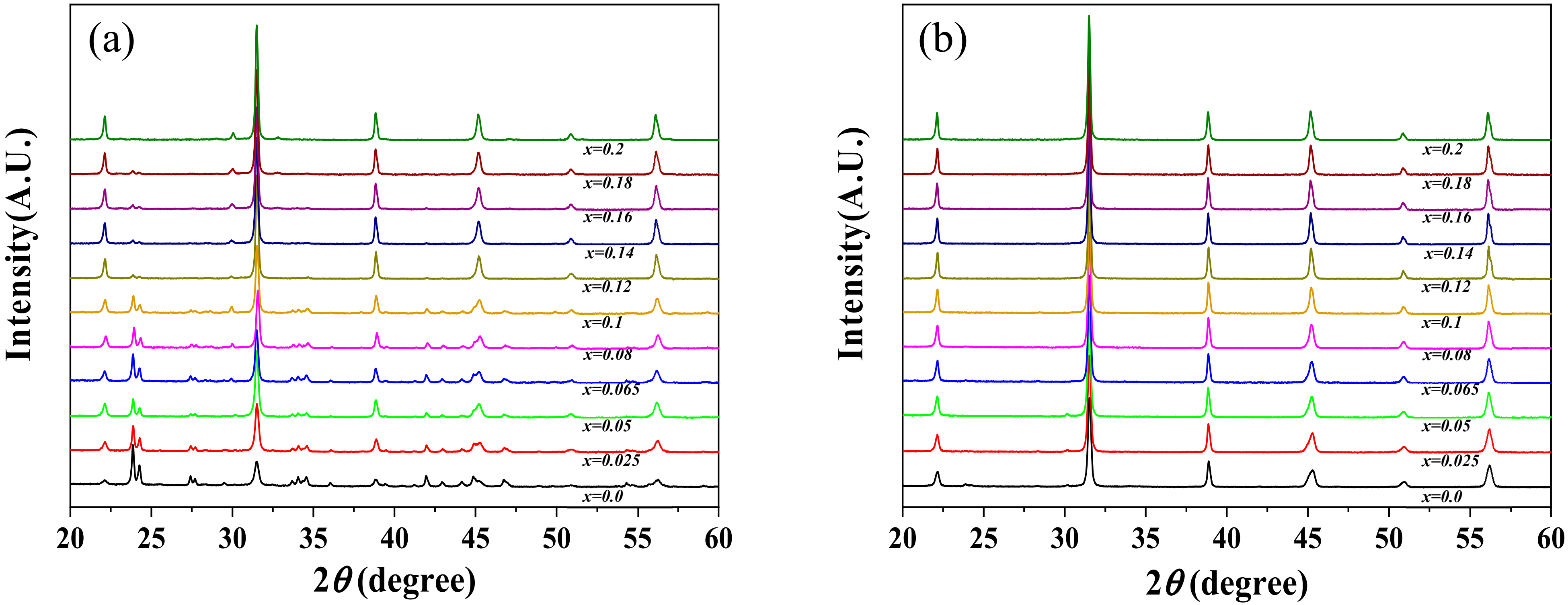

Figure S1. X-ray diffraction patterns for the (1-*x*)BT-*x*BZT samples (a) after the first calcination at 850 °C for 5 hours, and (b) after the second calcination at 950 °C for 2 hours. The micrometer-sized $BaTiO_3$ powder (< 2 µm, Sigma Aldrich) were used as a starting material.

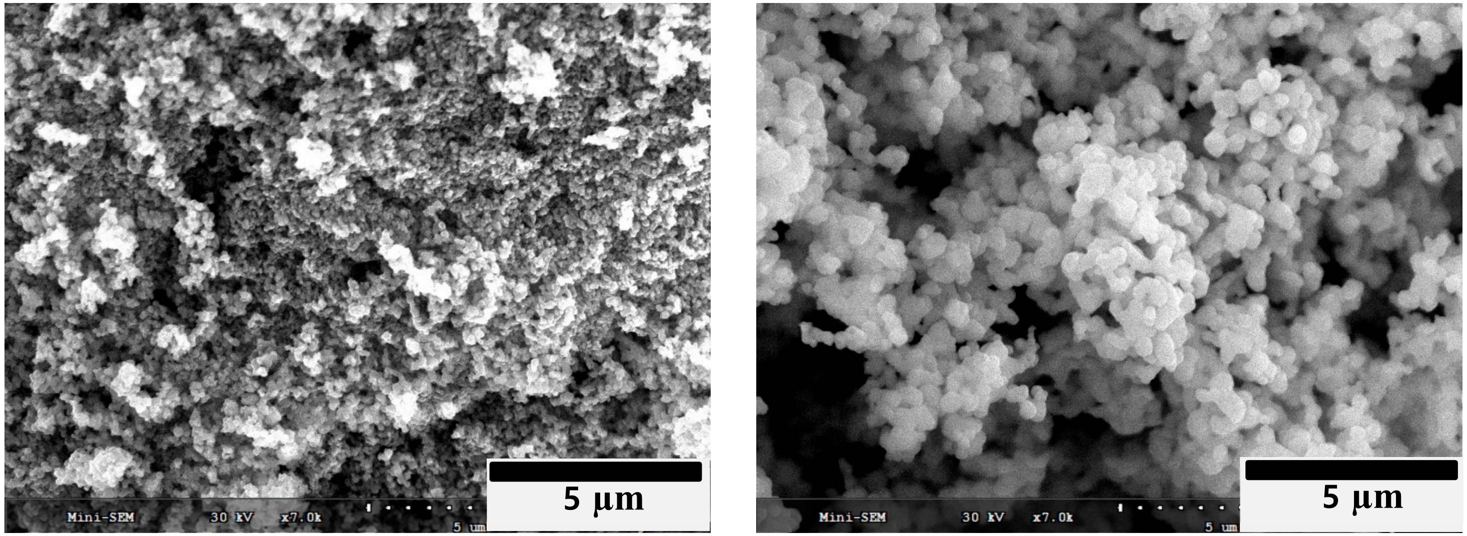

(a) (b)

Figure S2 Morphologies of the calcined powder: (a) nano-samples and (b) micro-samples.

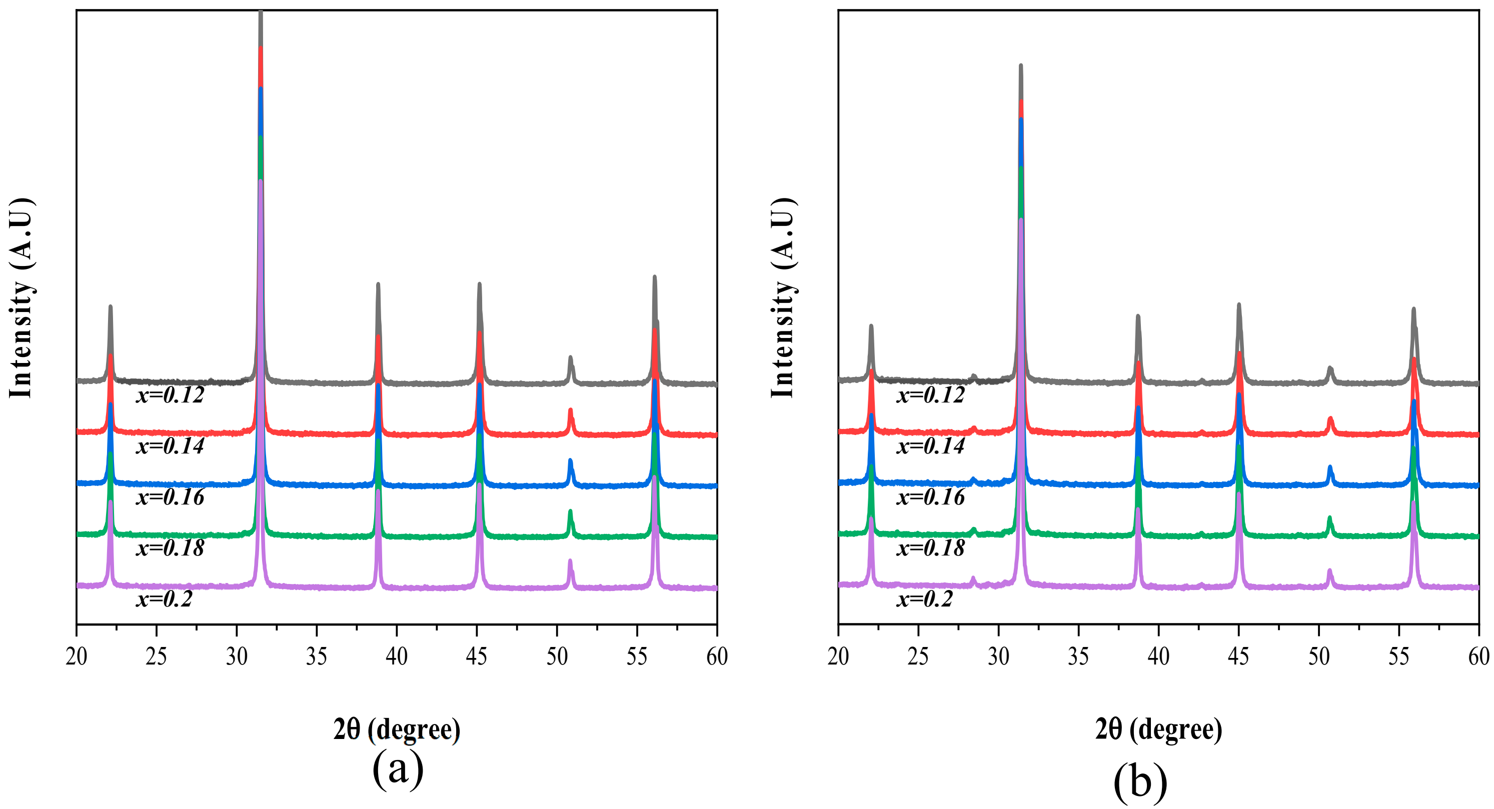

Figure S3 The XRD patterns of the sintered (1-*x*)BT-*x*BZT ceramics ($0.1 < x \leq 0.2$): (a) nano-samples and (b) micro-samples

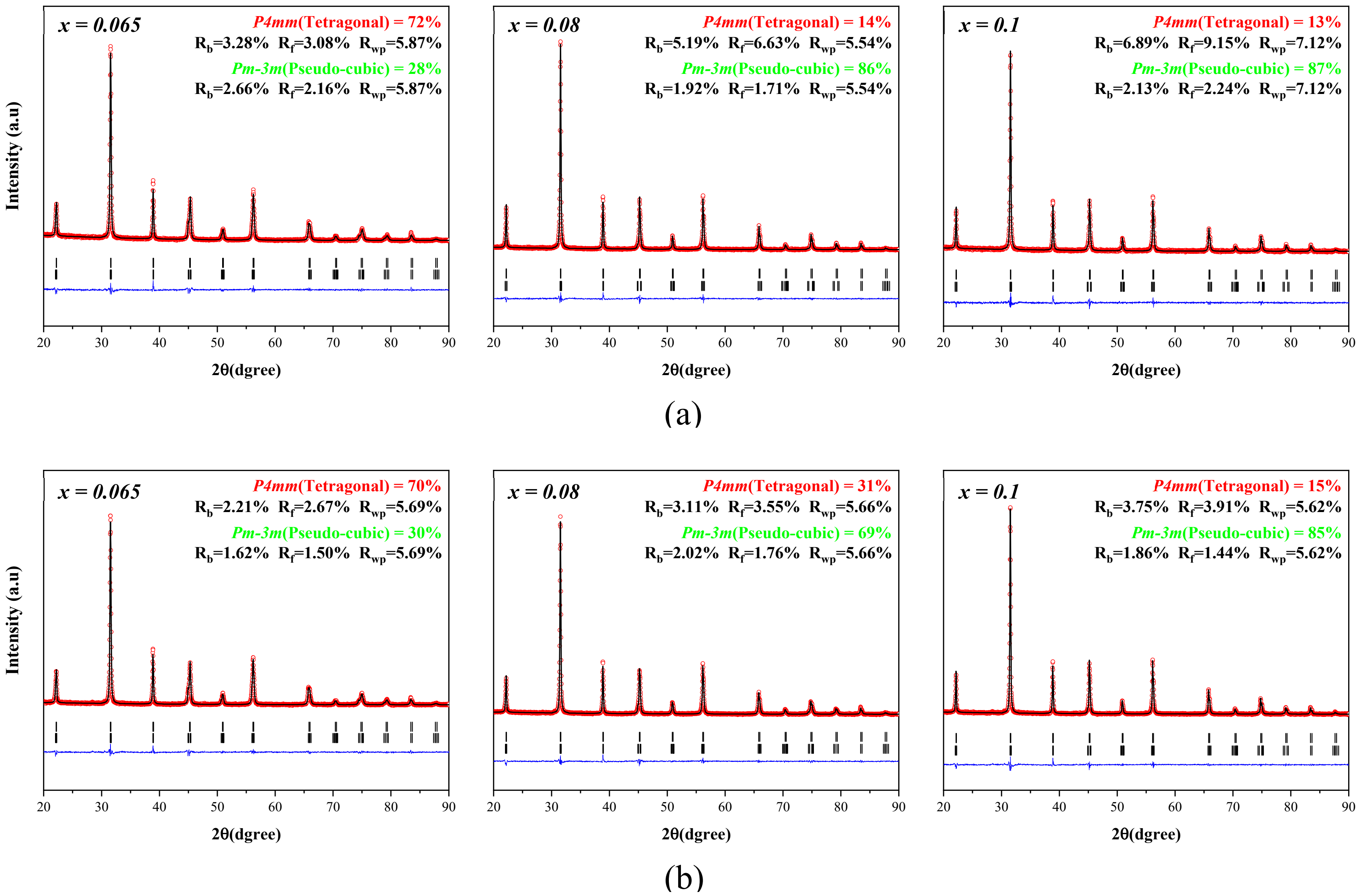

Figure S4 Rietveld refinement profiles for (1-*x*)BT-*x*BZT ceramics: (a) nano-samples and (b) micro-samples

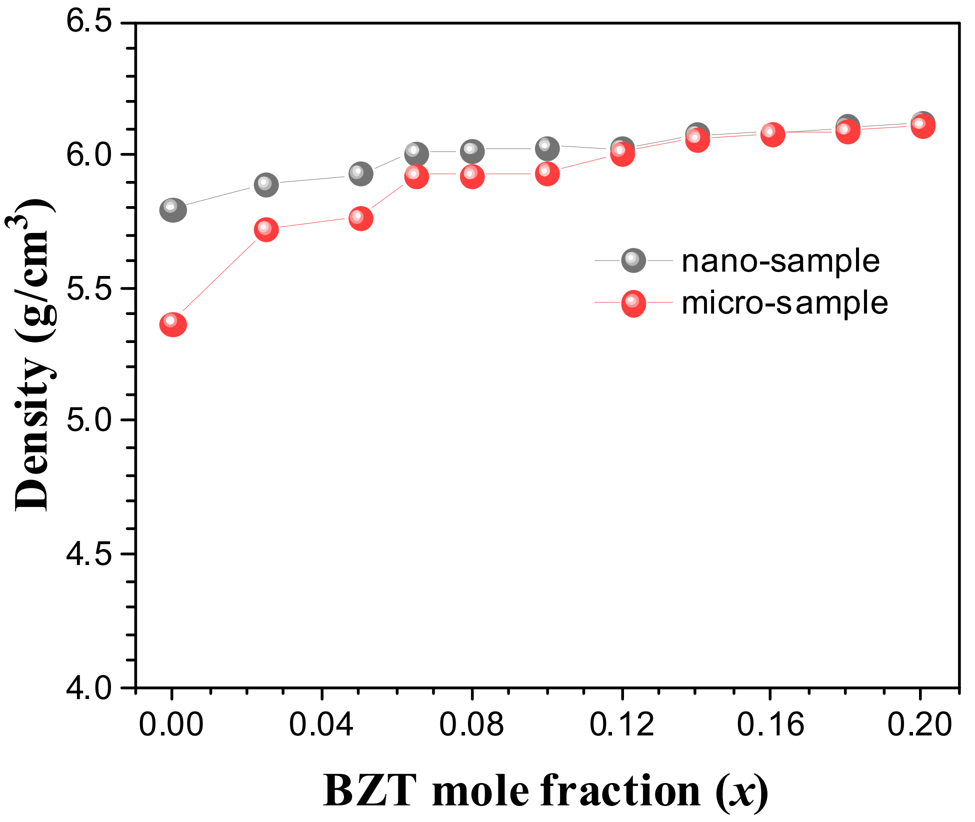

Figure S5 The densities of the sintered (1-*x*)BT-*x*BZT ceramics as a function of the BZT mole fraction.

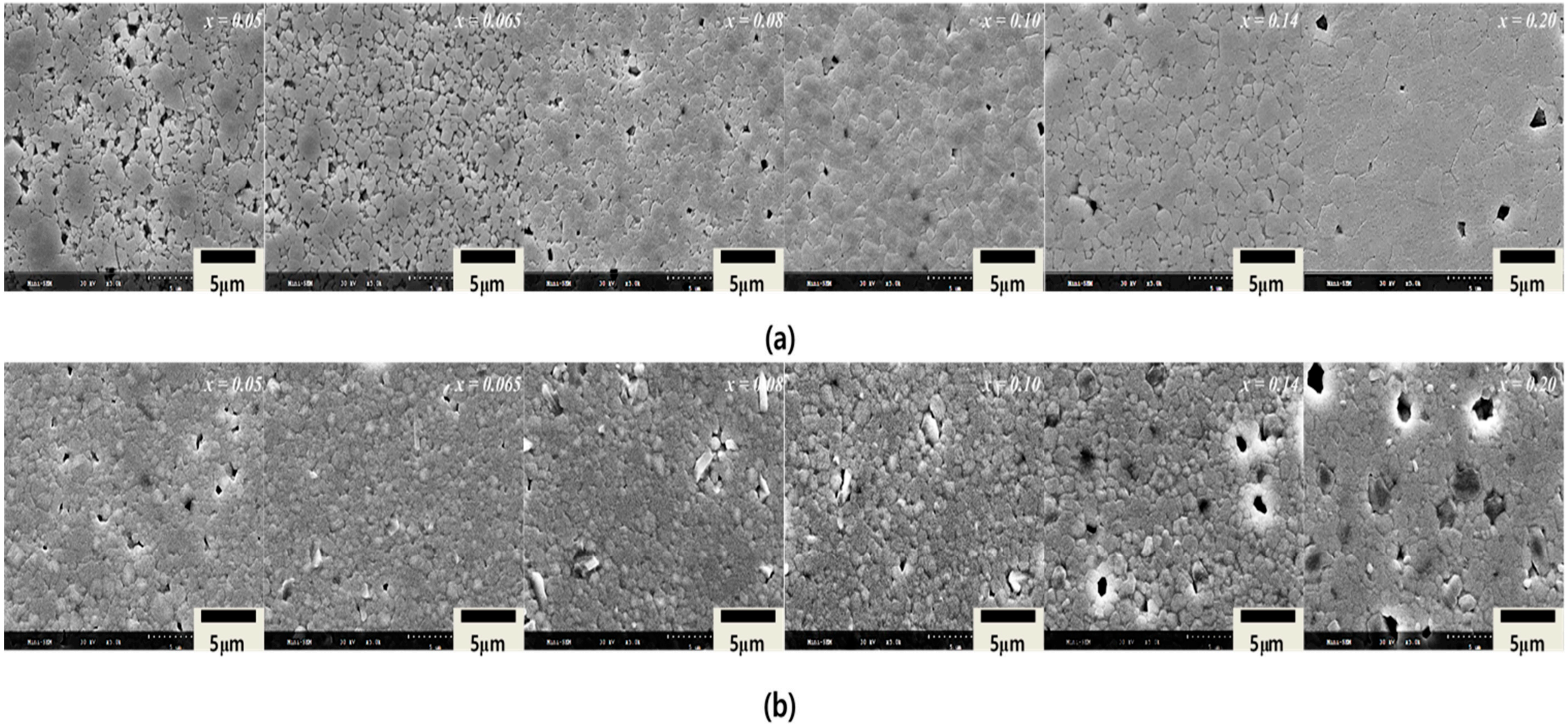

Figure S6 Microstructures of the (1-*x*)BT-*x*BZT ceramics: (a) nano-samples and (b) micro-samples.

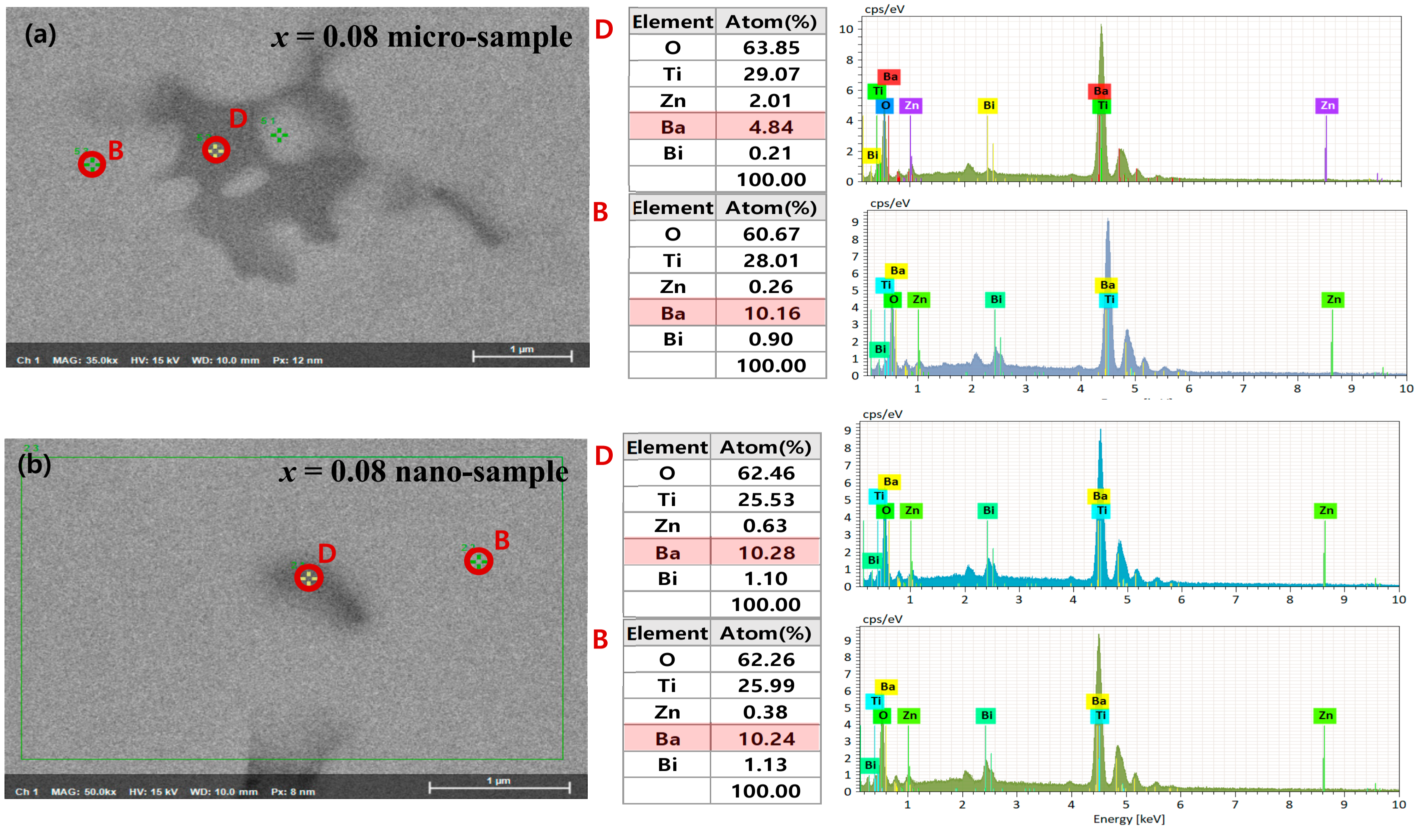

(a) D

Element	Atom(%)
O	63.85
Ti	29.07
Zn	2.01
Ba	4.84
Bi	0.21
	100.00

(a) B

Element	Atom(%)
O	60.67
Ti	28.01
Zn	0.26
Ba	10.16
Bi	0.90
	100.00

(b) D

Element	Atom(%)
O	62.46
Ti	25.53
Zn	0.63
Ba	10.28
Bi	1.10
	100.00

(b) B

Element	Atom(%)
O	62.26
Ti	25.99
Zn	0.38
Ba	10.24
Bi	1.13
	100.00

Figure S7 BSE images and EDS point analysis for a heterogeneous area with a dark contrast (D) and a matrix area with a bright contrast (B) **in** (1-x)BT-xBZT ceramics: (a) x = 0.08 micro-sample, (b) x = 0.08 nano-sample, (c) x = 0.20 micro-sample, and (d) x = 0.20 nano-sample.

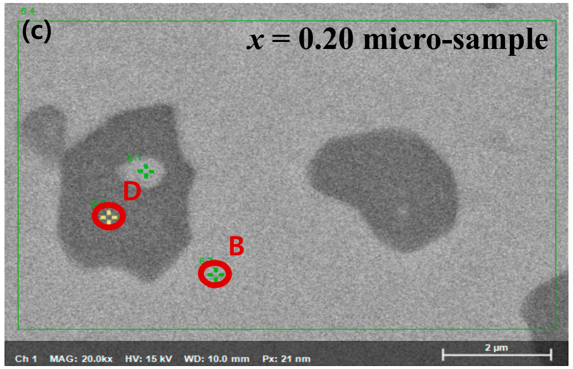

D

Element	Atom(%)
O	57.43
Ti	34.61
Zn	3.58
Ba	4.27
Bi	0.11
	100.00

B

Element	Atom(%)
O	49.32
Ti	31.35
Zn	2.06
Ba	12.86
Bi	4.41
	100.00

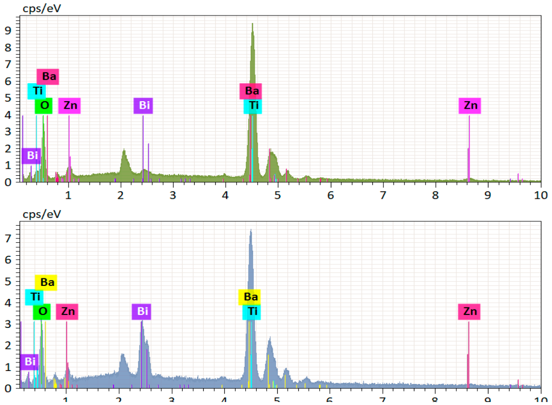

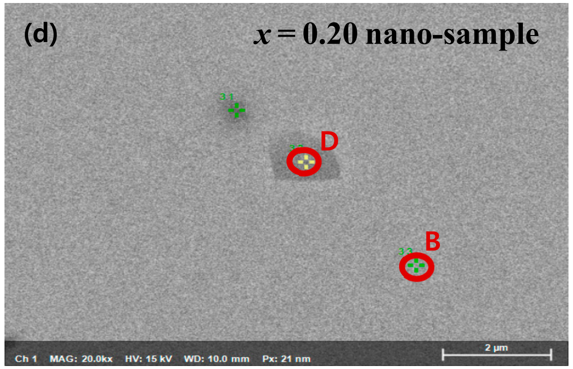

D

Element	Atom(%)
O	67.62
Ti	18.92
Zn	0.66
Ba	10.95
Bi	1.85
	100.00

B

Element	Atom(%)
O	59.79
Ti	26.77
Zn	1.05
Ba	9.97
Bi	2.42
	100.00

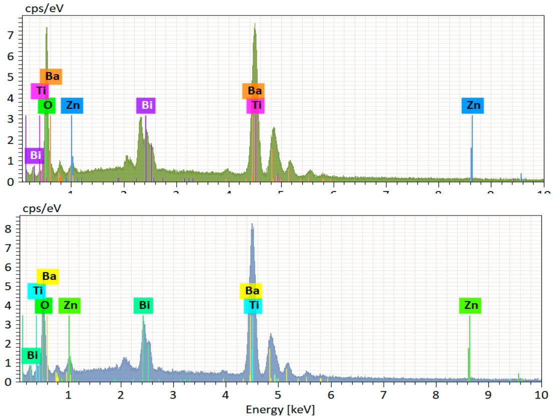

Figure S8 BSE images and EDS point analysis for a heterogeneous area with a dark contrast (D) and a matrix area with a bright contrast (B) in (1-x)BT-xBZT ceramics: (a) x = 0.08 micro-sample, (b) x = 0.08 nano-sample, (c) x = 0.20 micro-sample, and (d) x = 0.20 nano-sample.

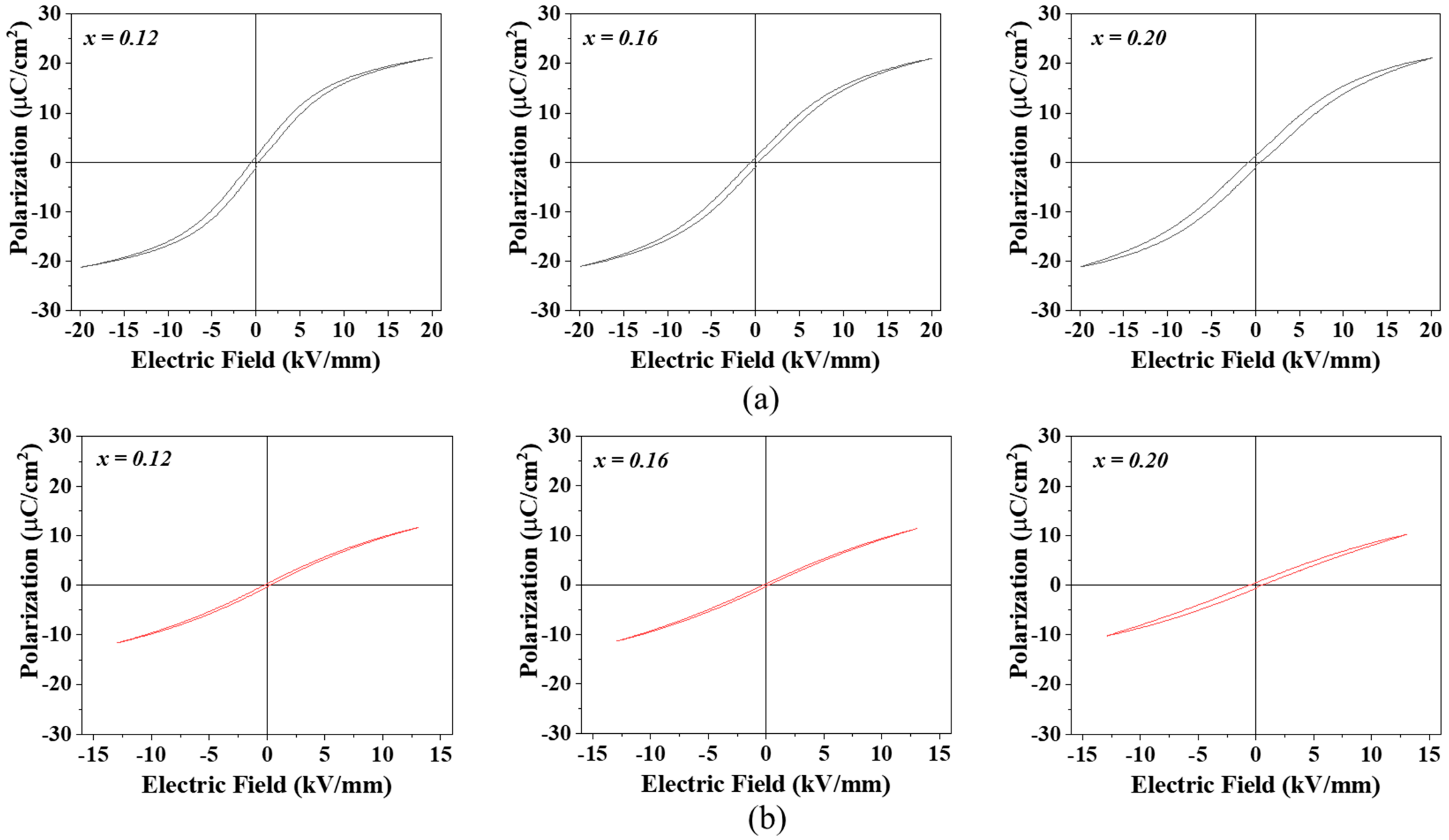

Figure S9 The P-E hysteresis curves of (1-x)BT-xBZT ceramics measured at room temperature (x = 0.12 ~ 0.20) by applying 20 kV/mm to the nano-samples and 13 kV/mm to micro-samples: (a) nano-samples and (b) micro-samples.

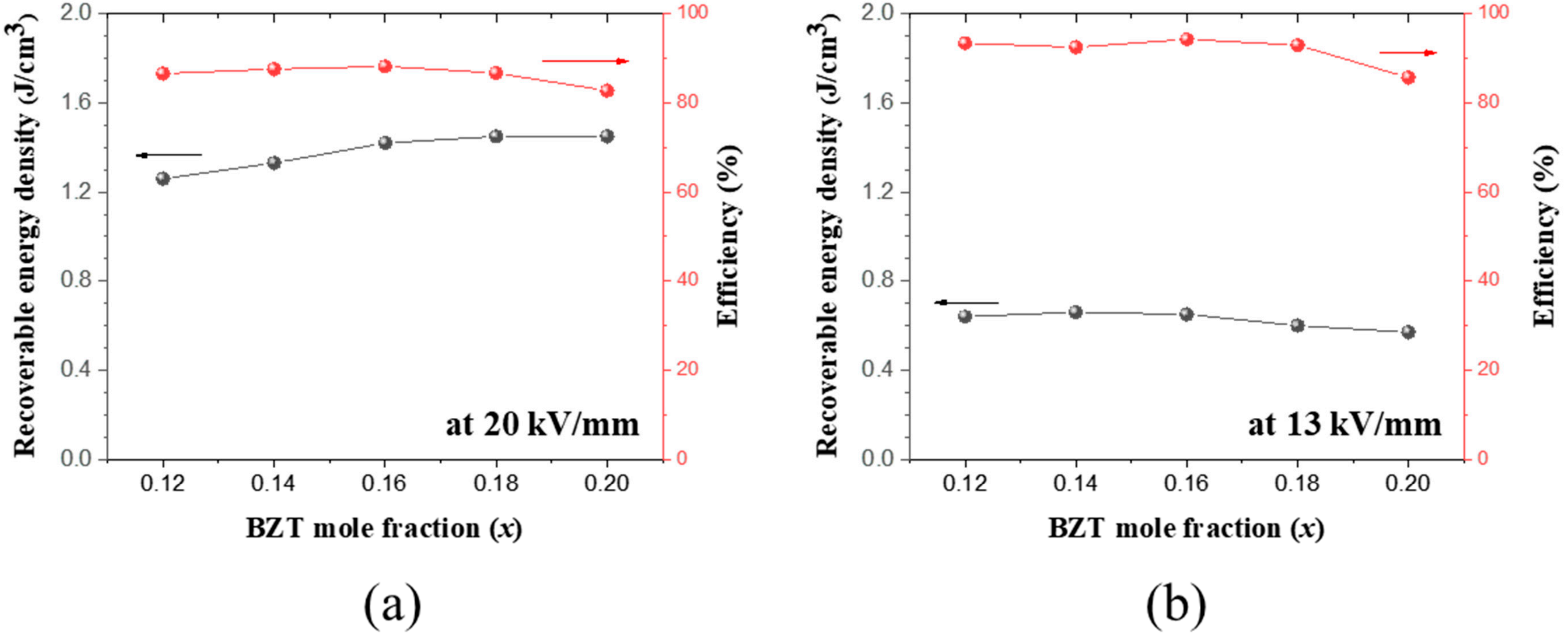

Figure S10 Recoverable energy density and discharge-to-charge energy efficiency as a function of the BZT mole fraction in the (1-*x*)BT-*x*BZT ceramics ($x = 0.12 \sim 0.20$): (a) nano-samples and (b) micro-samples.
